# Opipramol Inhibits Lipolysis in Human Adipocytes without Altering Glucose Uptake and Differently from Antipsychotic and Antidepressant Drugs with Adverse Effects on Body Weight Control

**DOI:** 10.3390/ph13030041

**Published:** 2020-03-05

**Authors:** Christian Carpéné, Francisco Les, Josep Mercader, Saioa Gomez-Zorita, Jean-Louis Grolleau, Nathalie Boulet, Jessica Fontaine, Mari Carmen Iglesias-Osma, Maria José Garcia-Barrado

**Affiliations:** 1 Institute of Metabolic and Cardiovascular Diseases, INSERM, UMR1048, Team 1, 31432 Toulouse, France; nathalie.boulet@inserm.fr (N.B.); jessica.fontaine@inserm.fr (J.F.); 2 I2MC, University of Toulouse, UMR1048, Paul Sabatier University, 31432 Toulouse, France; 3 Department of Pharmacy, Faculty of Health Sciences, Universidad San Jorge, 50830 Villanueva de Gállego Zaragoza, Spain; fles@usj.es; 4 Instituto Agroalimentario de Aragón-IA2, CITA-Universidad de Zaragoza, 50013 Zaragoza, Spain; 5 Department of Fundamental Biology and Health Sciences, University of the Balearic Islands, 07122 Palma, Spain; josep.mercader@uib.es; 6 Balearic Islands Health Research Institute (IdISBa), 07120 Palma, Spain; 7 Nutrition and Obesity Group, Department of Nutrition and Food Science, University of the Basque Country (UPV/EHU) and Lucio Lascaray Research Institute, 48940 Vitoria, Spain; saioa.gomez@ehu.eus; 8 Department of Plastic Surgery, CHU Rangueil, 31059 Toulouse, France; grolleau.jl@chu-toulouse.fr; 9 Laboratory of Neuroendocrinology, Institute of Neurosciences of Castilla y León (INCyL), University of Salamanca, 37007 Salamanca, Spain; mcio@usal.es (M.C.I.-O.); barrado@usal.es (M.J.G.-B.); 10 Laboratory of Neuroendocrinology and Obesity, Institute of Biomedical Research of Salamanca (IBSAL), University of Salamanca, 37007 Salamanca, Spain; 11 Department of Physiology and Pharmacology, Faculty of Medicine, University of Salamanca, 37007 Salamanca, Spain

**Keywords:** adipose tissue, obesity, monoamine oxidase, semicarbazide-sensitive amine oxidase, glucose transport, lipotoxicity, insulin, antidepressant, antipsychotic, harmine, phenelzine, opipramol

## Abstract

Treatment with several antipsychotic drugs exhibits a tendency to induce weight gain and diabetic complications. The proposed mechanisms by which the atypical antipsychotic drug olanzapine increases body weight include central dysregulations leading to hyperphagia and direct peripheral impairment of fat cell lipolysis. Several investigations have reproduced in vitro direct actions of antipsychotics on rodent adipocytes, cultured preadipocytes, or human adipose tissue-derived stem cells. However, to our knowledge, no such direct action has been described in human mature adipocytes. The aim of the present study was to compare in human adipocytes the putative direct alterations of lipolysis by antipsychotics (haloperidol, olanzapine, ziprazidone, risperidone), antidepressants (pargyline, phenelzine), or anxiolytics (opipramol). Lipolytic responses to the tested drugs, and to recognized lipolytic (e.g., isoprenaline) or antilipolytic agents (e.g., insulin) were determined, together with glucose transport and amine oxidase activities in abdominal subcutaneous adipocytes from individuals undergoing plastic surgery. None of the tested drugs were lipolytic. Surprisingly, only opipramol exhibited substantial antilipolytic properties in the micromolar to millimolar range. An opipramol antilipolytic effect was evident against isoprenaline-, forskolin-, or atrial natriuretic peptide-stimulated lipolysis. Opipramol did not impair insulin activation of glucose transport but inhibited monoamine oxidase (MAO) activity to the same extent as antidepressants recognized as MAO inhibitors (pargyline, harmine, or phenelzine), whereas antipsychotics were inefficient. Considering its unique properties, opipramol, which is not associated with weight gain in treated patients, is a good candidate for drug repurposing because it limits exaggerated lipolysis, prevents hydrogen peroxide release by amine oxidases in adipocytes, and is thereby of potential use to limit lipotoxicity and oxidative stress, two deleterious complications of diabetes and obesity.

## 1. Introduction

Many of the pharmaceutical agents prescribed to treat neuropsychiatric disorders exert metabolic effects. Several antipsychotic drugs, especially those associated with weight gain in treated schizophrenic patients [1], have been evidenced to enhance lipid storage in adipocytes [2]. However, it remains poorly determined whether extra-neuronal effects on adipocytes contribute to the unwanted fattening occurring during treatment, and even a few studies have reported that, at therapeutic concentrations, various antipsychotic agents cannot directly influence adipocyte metabolism [3]. The weight gain could therefore be a side-effect mainly triggered by central disturbances, such as increased food intake, leading to excessive calorie intake, then fattening, than by a direct activation of lipid storage [4,5]. In addition, it remains unclear whether it is the pharmaceutical treatment or the health recovery of the treated patient that influences food consumption. Nevertheless, numerous reports have indicated that various psychoactive agents are capable of directly modulate lipid accumulation in fat cells during short-term in vitro experiments. This is the case, but in the opposite sense, for an antidepressant drug, phenelzine, which inhibits insulin-induced lipogenesis in fat cells in less than an hour [6], impairs adipogenesis in cultured cells in the range of several days [7], and limits fat deposition in rodents chronically treated for weeks [8,9]. Indeed, the demonstration of a direct effect of a given psychoactive drug on adipocytes does not prevent the molecule to act in a concerted manner on gene regulation, and elsewhere on other cell types (and vice versa).

Due to these considerations stated, it appears of utmost importance to further explore whether adipose tissue is a site of action of antipsychotic drugs, especially those triggering metabolic dysregulation (e.g., olanzapine). In fact, the accumulation/breakdown of triglycerides in the lipid droplet of fat cells is rapidly regulated by neuronal, endocrine, and paracrine inputs, and is sensitive to many pharmacological agents. Previous investigations have been performed by measuring adipocyte biological functions and determining the regulations of key genes involved in metabolic pathways, either in adipocytes from treated animal models [10], or after short- or long-term incubation of cultured adipose cells with psychoactive molecules. To briefly mention examples of such different approaches, it is worth mentioning that the decreased lipolytic effect of isoprenaline and down-regulated hormone-sensitive lipase gene (*Hsl*) were found in adipocytes from olanzapine-treated rats [10], whereas increased lipid accumulation and adipogenic gene upregulation were observed in cultured adipocytes treated with olanzapine [2,11,12,13,14]. In this view, the reduction of the rates of lipolysis in response to the β-adrenergic agonist isoprenaline was consistently reported in both rats [10] and cultured preadipocytes [14] treated with olanzapine. Moreover, atypical antipsychotics have been described to alter other biological functions of adipocytes, not only related with the management of triglyceride storage/breakdown (lipogenesis and lipolysis) [11,15], but also related to insulin sensitivity and inflammation, such as adipokine secretion [12,14], therefore favoring the onset of insulin resistance. Nevertheless, no functional studies of the lipolytic responses to neuropsychiatric drugs have been performed in human mature adipocytes to date, whereas it has been documented that several antipsychotic drugs alter in vitro gene expression in human mesenchymal stem cells differentiated into adipocytes [2,16,17,18]. Besides focusing interest on lipolysis regulation, exploring the functional responses of freshly isolated human fat cells gives a greater preclinical interest to the reported pharmaceutical properties than observations performed on animal models or cell cultures.

Thus, in an attempt to obtain further insights on the direct effects of antipsychotic drugs on triglyceride breakdown in human adipocytes, we included olanzapine in the present comparative study for the evident following reasons. Olanzapine is a second-generation antipsychotic drug recognized for its metabolic side-effects in humans, leading to overweight and diabetes [4]. Rodents have provided valuable information about the olanzapine-induced metabolic adverse events, and a gender preferential overweight has been reported in female rats [10,19]. Olanzapine actions encompass mid-term regulations of gene expression [18], adipose tissue hypertrophy during prolonged treatment in rats [10,19], and even multigenerational alteration of glucose homeostasis in mice [20]. However, to our knowledge, only scarce information is available regarding olanzapine action on human adipocytes [17].

Other atypical antipsychotics such as ziprazidone or risperidone, claimed to develop less deleterious side-effects, were included in our comparative approach, especially when considering that fat stores can accumulate such lipophilic antipsychotic drugs up to 30 times higher than their therapeutic plasma levels [21]. Haloperidol was also tested as a reference of first-generation antipsychotics, exhibiting less “obesogenic” actions. We also included in this study several other psychoactive agents such as the antidepressant and neuroprotective agent phenelzine [22] in view of its above-mentioned inhibitory effects on lipid deposition.

Opipramol was prescribed for the treatment of anxious depression in the 1960s; it is a tricyclic antidepressant that does not inhibit the neuronal uptake of norepinephrine and/or serotonin [23]. Instead, it is considered as a weak dopaminergic, histaminergic, and serotoninergic antagonist, and binds to sigma receptors in the brain [23]. Our tests on mature adipocytes were therefore also extended to opipramol, as it is a psychotropic drug belonging to the classes of antidepressants, antipsychotics, and anxiolytics, but is not clearly associated with adverse effects affecting body weight regulation.

Our aim was to explore the short-term effects of the antipsychotics influencing body weight, as well as related anxiolytic, antiepileptic, or antidepressant drugs, on functional responses of human fat cells. Alongside the test of their lipolytic and antilipolytic capacities on freshly isolated adipocytes, we also studied their putative interactions with the amine oxidases expressed in adipocytes. Exploring the effects on monoamine oxidase (MAO) was driven by the similarities between the adipose (mainly of the A form in human adipocytes [24]) and the brain forms, well known to be the target of a class of antidepressants, the MAO inhibitors, which have been recently reported to mitigate excessive fat deposition in rodents [8,9,25,26]. Thus, pargyline, phenelzine, and harmine were used as representatives of MAO inhibitors in both assays of lipolytic or amine oxidation activities. Our approach also encompassed the study of putative interplay with the semicarbazide-sensitive amine oxidase (SSAO), highly expressed in human adipocytes [27], as several SSAO inhibitors (semicarbazide, phenelzine) are also endowed with potential anti-obesity properties [28]. Finally, for several of the above-mentioned pharmaceutical agents, our investigations compared their effects on hydrogen peroxide release by adipose tissue and on glucose transport because we have previously reported that amine oxidase substrates stimulate adipogenesis in cultured preadipocytes and glucose transport in adipocytes in a hydrogen peroxide-dependent manner [27].

The following results will indicate that, unexpectedly, opipramol behaved differently from the other psychoactive drugs in human adipose tissue by exhibiting substantial antilipolytic properties without altering glucose uptake capacities of human fat cells, and by exerting MAO inhibition.

## 2. Materials and Methods

### 2.1. Materials

Opipramol (4-[3-(5H-dibenz[b,f]-azepine-5-yl)-propyl]-1-piperazine-ethanol dihydrochloride), haloperidol, risperidone, phenelzine, pargyline, harmine, tyramine, benzylamine, semicarbazide, cytochalasin B, fatty acid-free bovine serum albumin, hydrogen peroxide, horseradish peroxidase, isoprenaline, cytochalasin B, and routinely used chemicals were obtained from Sigma-Aldrich-Merck (St. Quentin Fallavier, France), unless otherwise specified. Amplex Red was provided by InterChim (Montluçon, France). [^3^H]-2-Deoxyglucose, [^14^C]-benzylamine, and scintillation cocktail were purchased from PerkinElmer (Boston, MA, USA). Different [^14^C]-tyramine batches were either from PerkinElmer or Sigma. Olanzapine and ziprazidone were generous gifts from Prof. Renaud de Beaurepaire (Hosp. Paul-Guiraud, Villejuif, France).

### 2.2. Subjects and Preparation of Adipose Cells

Samples of human subcutaneous adipose tissue (hScAT) were obtained from patients undergoing abdominal lipectomy at the plastic surgical department of the Rangueil Hospital (CHU Toulouse, France) in accordance with the local ethic committee and French National Institute of Health (INSERM) guidelines under the agreement reference DC-2008-452. The adipocyte preparations used in the present study were obtained from a total of 26 healthy individuals (24 women) of a mean age of 39 years with a mean body mass index of 25.1 ± 0.8 kg/m^2^. As freezing/thawing sequences do not apply for obtaining functional adipocytes, the fat cells were immediately isolated by collagenase digestion as previously described [27]. Once cut into small pieces with scissors, portions of adipose tissue samples were digested at 37 °C under agitation. Meanwhile remaining portion of the samples was immediately frozen free from buffer at −80 °C in 35 mL plastic vials for subsequent homogenate preparation and determination of amine oxidase activities. Filtration of the digested pieces through nylon stockings and two washes of the buoyant fat cells allowed us to obtain preparations of functional adipocytes in Krebs-Ringer buffer with 15 mM bicarbonate, 10 mM 4-(2-hydroxyethyl)-1-piperazineethanesulfonic acid and 3.5% of bovine serum albumin (pH 7.4). A total of 6 mM glucose was added to this medium for lipolysis assays, but omitted for glucose uptake assays, performed with only the non metabolisable analog 2-deoxy-glucose as already reported [29].

### 2.3. Lipolysis Assays

Adipocyte suspension was distributed in plastic vials containing 400 µL and incubated at 37 °C under shaking (150 cycles/min) in the absence (basal) or in the presence of the indicated agents. After 90 min, the incubation tubes were placed on ice and 150 µL of cell medium was removed for the enzymatic determination of glycerol, as described previously [29]. Results were expressed as micromoles of glycerol released per 100 mg cell lipid for 90 min, as in [30]. As glycerol is an end-product of lipolysis less re-used than free fatty acids by human adipocytes, its release served as an index of lipolytic activity throughout the study, as in [31].

### 2.4. Oxidation of Radiolabeled Tyramine and Benzylamine by Adipose Tissue Homogenates

Human adipose tissue samples were thawed and homogenized at room temperature in 200 mM phosphate buffer (pH 7.5 for 30 s) to avoid the formation of a fat cake that is solid at cold temperatures and hampers enzymatic reactions. Amine oxidase activities were measured at room temperature with [^14^C]- tyramine or [^14^C]-benzylamine in 200 mM phosphate buffer, pH 7.5, for 30 min, as previously described [30]. The tested agents were preincubated for 15 min with homogenates prior the addition of substrate, which launched the reaction for 30 min, as in [32].

### 2.5. Hydrogen Peroxide Production by Adipose Tissue Homogenates

Activity of human amine oxidase was assessed by determining the hydrogen peroxide released by amine oxidation in homogenates of thawed hScAT. The same fluorimetric method was applied for MAO and for SSAO activity assays [33]. Hydrogen peroxide release was detected with a chromogenic mixture (4 U/mL horseradish peroxidase, 40 μM of fluorescent probe Amplex Red) in phosphate buffer, pH 7.5, and quantified with hydrogen peroxide standard curve (from 0.05 to 5 µM concentrations). Homogenates were distributed in 96-well dark microplates and incubated at 37 °C (200 µL/well) after pre-incubation without (control) or with the tested agents or 1 mM semicarbazide to abolish SSAO, or 1 mM pargyline to block MAO activity. Fluorescence data (ex/em: 540/590 nm) were collected on a Fluoroskan Ascent microplate reader (ThermoLabsystems, Finland), as in [34].

### 2.6. Glucose Transport Assays

After preincubation for 45 min at 37 °C with insulin, and/or indicated drugs, each vial (400 µL) received an isotopic dilution of the non-metabolizable hexose 2-deoxy-D-[3H]glucose, giving a final concentration of 0.1 mM (devoid of cold glucose, and equivalent to approximately 1,000,000 dpm per vial). Assays were then incubated for a 10 min period and stopped with 100 µL of 100 µM cytochalasin B. The radioactivity incorporated into the fat cells was separated by adding dinonyl phthalate (density 0.98 g/mL) and a pulse spin to microtubes containing 200 µL aliquots of cell suspension, as previously reported [29]. The upper part of the tubes, containing buoying adipocytes, were cut and counted in scintillation vials, as previously described [35].

### 2.7. Data Presentation and Statistical analyses

Experimental data are given as mean ± standard error of the mean (SEM) of the indicated number of independent observations, presented as histograms generated with Microsoft Excel and Powerpoint 14.6 for Mac or curves processed with GraphPad Prism 8.0, were analyzed by one-way ANOVA or Student’s *t*-test when appropriate. Statistical significance was assumed when *p* < 0.05; NS: not significantly different.

## 3. Results and Discussion

### 3.1. In Vitro Evaluation of the Direct Influence of Antipsychotics on the Lipolytic Responses of Human Adipose Cells

The lipolytic response of adipocytes freshly isolated from human subcutaneous fat depots represents a relatively simple model of assessing whether antipsychotic or antidepressant drugs can produce extra-neuronal effects, and thereby may alter body weight regulation or insulin sensitivity, besides altering satiety signals. Adipocyte lipolysis consists in an intracellular triglyceride breakdown leading to the release of glycerol and free fatty acids in the surrounding medium. Such biological function is finely regulated by lipolytic and antilipolytic modulators. Among the former, isoprenaline is a β-adrenergic agonist of reference (also known as isoproterenol), widely used for in vitro studies because it is capable of activating lipolysis to similar levels compared with the natural catecholamines epinephrine and norepinephrine, which activate both the lipolytic β- and antilipolytic α_2_-adrenoreceptors. Alongside isoprenaline, two other well-recognized lipolytic agents have been included in our study: forskolin, a direct activator of adenylyl-cyclase, and atrial natriuretic peptide (ANP), which activates guanylyl-cyclase in human adipocytes [36]. Insulin, the physiological antilipolytic hormone, was used as well as tyramine, a substrate of amine oxidases, for which we recently reported that its capacity to inhibit lipolysis is dependent on hydrogen peroxide production by human fat cells [31].

The influence of several antipsychotic agents was first tested on human adipocyte lipolysis under basal and isoprenaline-stimulated conditions. Basal lipolysis was not modified by haloperidol, representative of the first-generation antipsychotics, or by olanzapine, ziprasidone, and risperidone, which belong to the second-generation antipsychotics, at least when the drugs were incubated at 0.1 to 100 µM during 90 min with the adipocytes. All the drugs were unable to modify the basal value, which was 0.21 ± 0.03 µmoles of glycerol released per 100 mg cell lipid per 90 min, as they reached at 100 µM of the respective levels: haloperidol 0.26 ± 0.04, olanzapine 0.27 ± 0.04, ziprazidone 0.23 ± 0.03, and risperidone 0.22 ± 0.04 µmol/100 mg/90 min (*n* = 10–11; NS).

**Figure 1:**
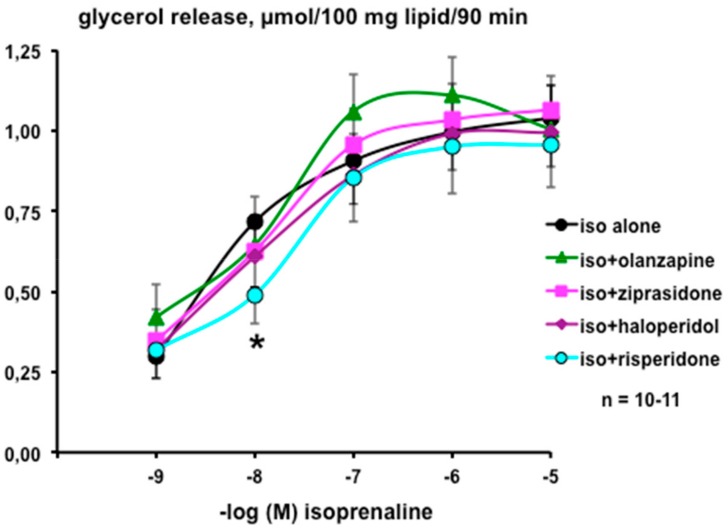
Dose–response curves for isoprenaline stimulation of lipolysis in human subcutaneous adipocytes in the presence of antipsychotic drugs. Each point is the mean ± SEM of 10–11 determinations, performed in control conditions with isoprenaline alone (iso alone, black circles) or in combination with 100 µM of the indicated drugs. A significant difference from the corresponding control lipolytic response was found only in the presence of risperidone (iso+risperidone, blue circles), at: * *p* < 0.05.

In contrast, isoprenaline clearly stimulated lipolysis in a dose-dependent manner, and this was not altered by the presence of 100 µM of the above-mentioned antipsychotics (Figure 1). No significant differences were observed between the respective EC_50_ values obtained from non-linear regression analysis of the dose response-curves: isoprenaline: 2.5 ± 0.5 nM; isoprenaline + ziprazidone: 4.6 ± 3.1 nM; isoprenaline + olanzapine: 4.6 ± 11.8 nM; isoprenaline + haloperidol: 4.7 ± 3.4 nM; isoprenaline + risperidone: 10.7 ± 13.4 nM. The only observed direct effect of these antipsychotics was that of risperidone, which impaired the submaximal effect of 10 nM isoprenaline without altering its maximal lipolytic effect (Figure 1).

These observations contrast with the reported rapid inhibition of basal lipolysis by ziprazidone in rat fat cells, but they confirm the lack of immediate action of haloperidol and olanzapine [10]. Our findings also contrast with the short-term effects of olanzapine, which at 100 µM inhibited isoprenaline-induced lipolysis in rat adipocytes, whereas risperidone was inefficient at the same dose [11]. Moreover, these observations are in line with the very recent findings of Sarsenbayeva et al., who reported during the completion of our work that olanzapine can lower basal lipolysis after short-term incubation (30 min) with human adipocytes, but not after longer treatment (24 h) [37]. Therefore, the fattening side-effect of olanzapine found in psychotic patients under prolonged treatment (Zyprexa^®^ at >15 mg/day) did not appear to be mediated by an immediate modulation of lipolytic activity in adipocytes. This does not mean that the effects of the drug are only central, as olanzapine can up-regulate or down-regulate gene expression of various key enzymes involved in the regulation of triglyceride synthesis/breakdown [10].

It was then verified whether these antipsychotics hamper the antilipolytic action of insulin. Unfortunately, the insulin sensitivity of human adipocyte preparations exhibited high inter-individual variability and signs of insulin resistance—100 nM of the pancreatic hormone tended to inhibit the 10 nM isoprenaline-induced lipolysis by only 21.3 ± 12.0% (*n* = 9; NS). Nevertheless, this percentage of inhibition (antilipolytic effect) raised to 23.6 ± 9.8%, 24.8 ± 12.2%, 39.0 ± 15.1%, and 44.7 ± 11.5% with 100 µM of olanzapine, ziprazidone, haloperidol, and risperidone, respectively (*n* = 9; NS). Thus, no dramatic alteration of insulin responsiveness was observed immediately after the application of antipsychotic drugs, at least when considering the blunted antilipolytic responses of overweight individuals. This was in agreement with the lack of change of the adipocyte insulin responsiveness of rats after prolonged administration of atypical antipsychotic drugs [10], and with the limited improvement of insulin antilipolytic effect during short-term treatments of rat adipocytes [10,11].

### 3.2. Influence of Opipramol on Basal and Stimulated Lipolytic Activity of Human Adipose Cells

We extended a similar approach to the atypical anxiolytic and antidepressant drugs opipramol (Ensidon) and phenelzine (Nardil) by performing lipolysis assays on other sets of adipocyte preparations. Our working hypothesis was that opipramol, which is an iminostilbene derivative, would not exhibit any deleterious effect on lipolysis because it is not recognized for causing weight gain in patients [23], and that phenelzine could be potentially lipolytic because we recently reported that this MAO inhibitor limits fat accumulation in rodents [8,9,25], a finding observed with selegiline, another MAO inhibitor [26].

**Figure 2:**
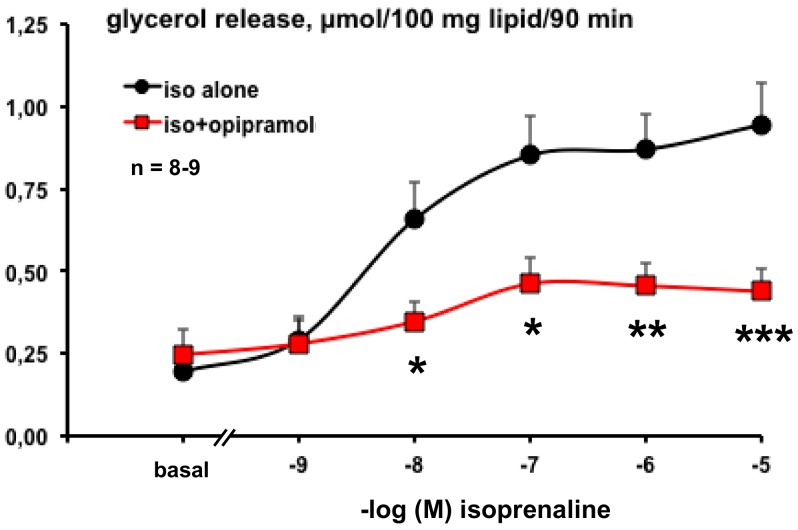
Impairment by opipramol of isoprenaline lipolytic activation in human subcutaneous adipocytes. Isoprenaline was tested alone at increasing concentrations (iso alone, black circles, control) or in the presence of 100 µM opipramol (red squares). Spontaneous glycerol release from freshly isolated adipocytes (basal) was also tested without and with opipramol. Each point is the mean ± SEM of 8–9 determinations. Different from the corresponding control at *: *p* < 0.05; **: *p* < 0.01; ***: *p* < 0.001.

A serendipitous observation was that opipramol dramatically inhibited isoprenaline activation of lipolysis in human adipocytes. The sigmoid dose–response to the β-adrenergic agonist (characterized by an IC_50_ of 3.3 ± 1.9 nM) was flattened in the presence of 100 µM opipramol (Figure 2).

**Figure 3:**
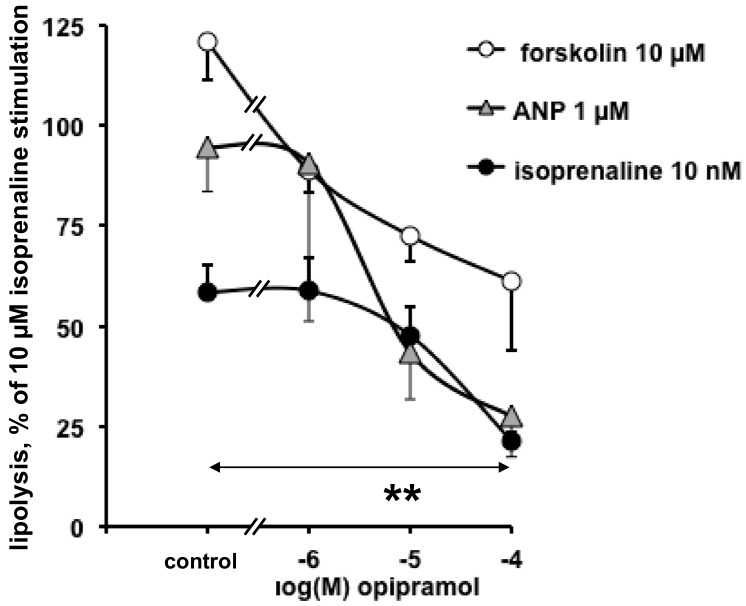
Opipramol exhibited inhibitory effect on various lipolytic agents in human abdominal adipocytes. The lipolytic responses to 10 µM forskolin, 1 µM atrial natriuretic peptide (ANP), or 10 nM isoprenaline were expressed as percentage of the maximal glycerol release capacity (which reached 0.9 ± 0.1 µmol glycerol/100 mg lipid/90 min in response to 10 µM isoprenaline). Each of these responses with lipolytic agent alone (control) was impaired by increasing doses of opipramol (from 1 to 100 µM). Each point is the mean ± SEM of six different adipocyte preparations. Difference between 100 µM opipramol and its corresponding control with each lipolytic agent alone at **: *p* < 0.01.

Opipramol is known to bind with high affinity to sigma receptors and to a lesser extent to dopamine receptors [38], but its putative affinity for β-adrenergic receptors is poorly documented. We tested whether such an unexpected antilipolytic effect of opipramol was also observed when lipolysis is activated by lipolytic agents other than isoprenaline, in order to discard a putative antagonism at β-adrenergic receptors. Figure 3 shows that increasing doses of opipramol impaired the lipolytic effect of forskolin (a direct activator of cAMP generation by adenylyl-cyclase), and the cGMP-dependent stimulatory effect of ANP as well. Figure 3 also shows that the inhibition of the submaximal activation of lipolysis by 10 nM isoprenaline was clearly dose-dependent for opipramol between 1 and 100 µM (the IC_50_ values ranged around 7 µM).

However, in our conditions, the dose of 1.0 µM opipramol was too low to produce a clear-cut antilipolytic action, irrespective of the lipolytic agent used to activate triglyceride breakdown. Because the therapeutic plasma levels of this anxiolytic drug are estimated as being around 0.1 µM, the antilipolytic effects of opipramol are relevant only in case of overdose or if the drug is accumulated in adipose tissue [39]. This might explain why opiramole administration is not considered to induce weight gain in anxious patients. Nonetheless, our experiments indicated that, despite impairing the stimulation by various lipolytic agents, opipramol was not able to lower the baseline of glycerol release (see Figure 2).

Indeed, testing the putative antilipolytic properties of any given agent in basal conditions is poorly relevant. This may explain some of the discrepancies found in the literature regarding the effects of antipsychotic and antidepressant drugs on basal lipolysis [10,11,37]. To further characterize any antilipolytic capacity, it is more discerning to work under prestimulated conditions. This was therefore performed by testing how the antilipolytic effect of opipramol could be improved or impaired by other known antilipolytic agents, such as insulin or tyramine, on isoprenaline submaximal stimulation. As opipramol augments the therapeutic action of neuroleptics, it was also tested as to whether its antilipolytic effect was modulated by haloperidol, whereas our study also included the combination with other inhibitors.

**Figure 4:**
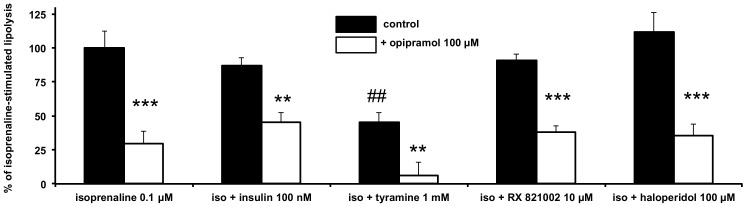
Combination of opipramol with insulin, tyramine, RX 821002, or haloperidol on isoprenaline-induced lipolysis in human adipocytes. Human subcutaneous fat cells were incubated for 90 min with 0.1 µM isoprenaline (iso) alone or in combination with the following agents: 0.1 µM insulin, 1 mM, tyramine, 10 µM RX 821002, 100 µM haloperidol. Results are expressed as percentage of 0.1 µM isoprenaline-induced glycerol release, which averaged 0.91 ± 0.11 µmol glycerol/100 mg lipid/90 min (*n* = 6). In parallel, adipocyte suspensions were also incubated in the presence of 100 µM opipramol. Mean ± SEM of six cases. The lipolysis was significantly lower in the presence of opipramol (open columns) than in respective control (control, black columns) at **: *p* < 0.01, ***: *p* < 0.001. Tyramine inhibited isoprenaline stimulation at ##: *p* < 0.01, whereas other agents were inefficient.

Again, insulin did not significantly inhibit isoprenaline-stimulated lipolysis in this second set of experiments, that is, when tested at 100 nM against equivalent dose of isoprenaline (Figure 4). This is not so inconsistent, as human adipocytes are recognized as being less insulin-sensitive than rodent fat cells, especially in the case of overweight individuals [40]. Nevertheless, the presence of opipramol did not alter the antilipolytic effect of insulin—there was neither worsening nor addition of their effects when the two agents were tested simultaneously. In contrast, millimolar concentration of tyramine was remarkably antilipolytic in human adipocytes, as previously reported [31], and its effect was added to that of opipramol when combined, leading to an almost complete blockade of isoprenaline lipolytic stimulation (Figure 4). The α_2_-adrenergic antagonist RX 821002 (also known as methoxy-idazoxan), already reported to inhibit the antilipolytic action of α_2_-agonists in human adipocytes [31], was unable to counteract the opipramol effect. Haloperidol did not hamper isoprenaline stimulation, in accordance with its poor effect on weight gain in treated patients [4], and with its above-reported lack of effect on lipolysis (see Figure 1), and it did not alter opipramol antilipolysis (Figure 4). Lastly, the opipramol antilipolytic effect also remained unchanged in the presence of the nitic oxide synthase inhibitors aminoguanidine and L-NAME (Ngamma-nitro-L-arginine methyl ester) (not shown). From these observations, a putative contribution of α_2_-adrenergic component in the antilipolytic effect of opipramol could be discarded, as well as any interaction with β-adrenergic receptors.

Regarding the effect of opipramol on its own on basal lipolysis, a lack of effect was evident from 1 to 100 µM (e.g., 0.18 ± 0.06 vs. 0.19 ± 0.06 µmol glycerol released/100 mg lipid/90 min for 10 µM opipramol and basal, respectively, *n* = 6; NS). In all, it could be definitively assessed that opipramol was not lipolytic, but the exact mechanism of action of its antilipolytic capacity, unmasked in prestimulated situations, could not be determined.

Because tyramine, a MAO substrate in human adipocytes [24,31], exhibited an effect additive to that of opipramol, it was necessary to verify whether the latter was interacting with MAO. Before such verification, we investigated the phenelzine short-term effects on lipolysis, as phenelzine is a well-recognized MAO inhibitor exhibiting both central effects [41] and direct negative control of lipid accumulation in adipocytes [6,7].

### 3.3. Influence of Phenelzine and Pargyline on Basal and Stimulated Lipolytic Activity of Human Adipose Cells

Because phenelzine mitigates excessive fat deposition in mice fed a high-fat diet [8,9], and directly impairs adipogenesis in cultured preadipocytes [7], we presumed that it could directly favor triglyceride breakdown in adipose cells.

When tested in similar conditions as above, phenelzine did not acutely alter the effect of isoprenaline. The maximal effect of 10 µM isoprenaline, set at 100%, was unaltered by the MAO inhibitor, as in the presence of 1 mM phenelzine it remained 85 ± 10% of the lipolytic stimulation (*n* = 7; NS). The historical antidepressant pargyline, another reference of MAO inhibitor, was also inactive at 1 mM (85 ± 6%; *n* = 7; NS). In addition, even when tested alone at low or high doses, phenelzine and pargyline were devoid of notable influence on basal lipolysis, representing at 1 mM 16 ± 10% and 10 ± 18% of maximal effect reached with 10 µM isoprenaline (*n* = 7; NS).

**Figure 5:**
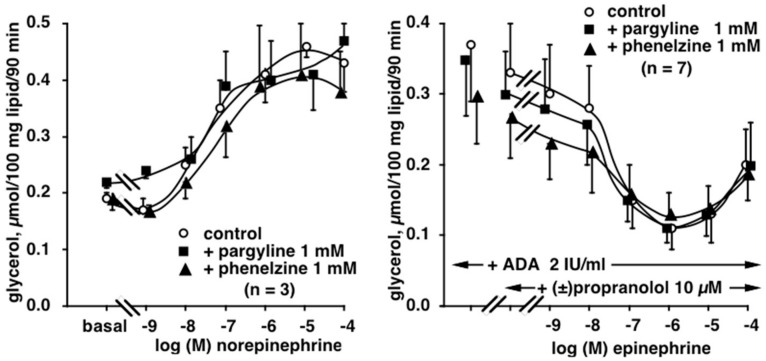
Lack of influence of monoamine oxidase (MAO) inhibitors pargyline and phenelzine on the norepinephrine and epinephrine effects on lipolysis in human subcutaneous adipocytes. Left panel: dose–response curves of glycerol release were obtained with norepinephrine alone (control, open circles) or in the presence of pargyline (squares) or phenelzine (triangles) at 1 mM. Mean ± SEM of three adipocyte preparations. No significant difference between control and 1 mM inhibitor. Right panel: biphasic action of epinephrine in the presence of propranolol and adenosine deaminase (ADA) in order to unmask the α2-adrenergic antilipolytic component of the catecholamine (control, open circles). Mean ± SEM of seven adipocyte preparations. No significant difference between control and 1 mM pargyline (squares) or phenelzine (triangles).

Considering that phenelzine and pargyline are known as MAO inhibitors, and that norepinephrine and epinephrine are MAO substrates whereas isoprenaline is not, we further tested both antidepressant drugs on (nor)epinephrine-stimulated lipolysis. The norepinephrine dose–response exhibited a classical sigmoid shape that was not altered by the MAO inhibitors (Figure 5, left panel). Although rather surprising, this finding seems to indicate that MAO inhibitors did not act as protective agents for norepinephrine, which is readily oxidized by MAO essentially at supra-micromolar doses. Indeed, at 1 µM, norepinephrine already reached its maximal activation of lipolysis, and any protection from its degradation cannot further enhance the amplitude of the maximal plateau, observed between 1 and 100 µM.

However, we hypothesized that catecholamine protection against MAO oxidation could be beneficial not only for the β-adrenergic lipolytic component, but also for the α_2_-adrenergic antilipolytic effect of (nor)epinephrine, as both β- and α-adrenoceptors bind these molecules. This was tested by determining the epinephrine-dependent inhibition of a mild lipolytic activation by adenosine deaminase (ADA) in the presence of the β-adrenoceptor antagonist propranolol. Effectively, under such β-blockade, epinephrine impaired the lipolytic effect of ADA, at least between 0.01 and 10 µM, as at 100 µM the β-adrenergic lipolytic component competed for β-adrenergic blockade by 10 µM propranolol and tended to re-increase glycerol release, resulting in a “U-shaped” curve (Figure 5, right panel). Again, both α_2_- and β-adrenergic components were unaltered in the presence of phenelzine or pargyline.

**Figure 6:**
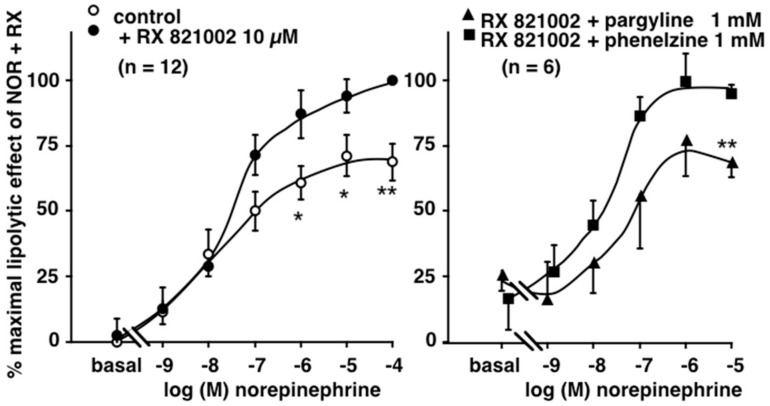
Influence of the MAO inhibitors pargyline and phenelzine on the potentiation of norepinephrine-induced lipolysis by α2-adrenergic blockade. Left panel: dose–response curves of glycerol release were obtained with norepinephrine alone (control, open circles) or in the presence of RX 821002 (NOR+RX, closed circles). Results are expressed as percentage of maximal response to NOR+RX. Mean ± SEM of 12 adipocyte preparations. Different from respective NOR+RX at *: *p* < 0.05; **: *p* < 0.01. Right panel: Influence of phenelzine (triangles) pargyline (squares) at 1 mM on the RX 821002-induced potentiation of norepinephrine lipolytic activation. Mean ± SEM of six cases. Different from NOR+RX at **: *p* < 0.01.

Another experiment aimed to verify whether pargyline or phenelzine could influence the lipolysis modulation by norepinephrine. It consisted in the addition of 10 µM RX 821002 to norepinephrine increasing doses, without and with MAO inhibitors. As expected, the blockade of the α_2_-adrenergic antilipolytic component of norepinephrine by RX 821002 enhanced its lipolytic effect, resulting from a predominant β-lipolytic pathway in the α_2_/β balance (Figure 6, left panel). Such observation was confirmatory of previous findings [42]. However, phenelzine did not further enhance the lipolytic action of norepinephrine, even when its α_2_-component was blocked (Figure 6, right panel). Unexpectedly, pargyline at a millimolar dose exhibited a tendency to partially blunt the potentiation of norepinephrine by RX 821002. None of these observations indicated that the MAO inhibitors could be qualified as lipolytic or pro-lipolytic agents. Obviously, they did not exhibit the noticeable antilipolytic effect observed with opipramol. Although the antidepressant phenelzine is recognized as inducing some weight gain in patients, this effect is of lower amplitude than with other antidepressants [41], and this is not supported by any higher activating influence on lipid mobilization when compared to the antipsychotics tested here. Because phenelzine has been reported to modestly limit fat accretion in animal models [8,9,25], this might rather be related to an impairment of lipogenic activity, as reported in mouse fat cells [6].

Because opipramol behaved differently from various other psychoactive substances, at least regarding lipolysis regulation in fat cells, we examined the way in which it interacted with MAO activity and another major amine oxidase expressed in adipose tissue—the semicarbazide-sensitive amine oxidase (SSAO) [43]. To this aim, putative interaction with MAO was determined on [^14^C]-tyramine oxidation by human adipose tissue homogenates, whereas the interaction with SSAO was tested by inhibition of [^14^C]-benzylamine oxidation.

### 3.4. Interactions of Opipramol and Other Psychotropes with Adipose Tissue Amine Oxidase

**Figure 7:**
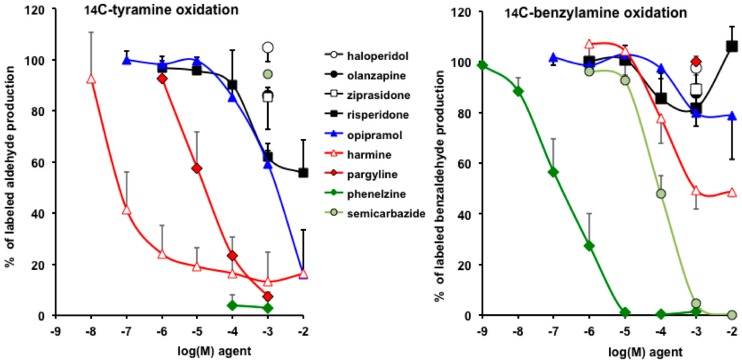
Influence of typical antipsychotics (haloperidol), atypical antipsychotics (olanzapine, ziprazidone, risperidone), MAO inhibitor antidepressants (phenelzine, pargyline), tricyclic antidepressants (opipramol), and various inhibitors (harmine, semicarbazide) on [^14^C]-tyramine and [^14^C]-benzylamine oxidation by human white adipose tissue. Homogenates (approximately 100 µg protein/100 µL) were preincubated for 15 min with the indicted agents, then incubated for 30 min in the presence of 0.5 mM [^14^C]-tyramine (left panel) or 0.5 mM [^14^C]-benzylamine (right panel). For each amine, results are expressed as respective oxidation without added agent (100%). Each point is mean ± SEM of four to nine observations.

To describe the interaction of opipramol and other psychoactive substances with peripheral MAO, we compared their capacity to inhibit the oxidation of 0.5 mM [^14^C]-tyramine by human adipose tissue homogenates (Figure 7, left panel). In parallel, competition experiments were performed with 0.5 mM [^14^C]-benzylamine (Figure 7, right panel), a SSAO substrate that produces H_2_O_2_ once oxidized, and this activates glucose uptake into human fat cells [27].

As expected, the MAO inhibitors pargyline and phenelzine totally abolished tyramine oxidation while semicarbazide was inactive, even at 1 mM. Notably, opipramol dose-dependently inhibited MAO activity with an IC_50_ that was higher than that of harmine, a MAO-A inhibitor of reference [44]. Risperidone also impaired tyramine oxidation, without reaching complete inhibition at 10 mM. Tested at a high dose only, the antipsychotics either typical (haloperidol) or atypical (olazanpine, ziprazidone) were totally unable to hamper MAO activity.

[^14^C]-Benzylamine oxidation was totally abolished by the reference SSAO inhibitor semicarbazide, whereas the MAO selective inhibitor pargyline was inefficient, as it was the case of the antipsychotics at supra-therapeutic dose. Opipramol slightly limited SSAO activity at 1–10 mM. The strongest inhibitor was phenelzine, which totally blocked SSAO activity, as did semicarbazide (respective IC_50_ were 0.6 ± 0.2 µM and 80 ± 12 µM) (Figure 7). This is in agreement with the novel nomenclature of SSAO, renamed PrAO, for primary amine oxidase [45], as the enzyme cannot be named further on the basis of its sensitivity to its historical inhibitor semicarbazide, which is currently overpassed by other molecules being more selective and potent (listed in [28]). Nevertheless, it seems that it is the first time that such an impressive capacity of phenelzine in inhibiting human SSAO is reported for a native, tissue-bound form (neither recombinant nor purified).

Figure 7 also shows a greater inhibition of the oxidation of tyramine than benzylamine by harmine, an historical inhibitor of MAO, able to limit the degradation of tyramine, serotonin [46], and kynuramine [44], that is, having more selectivity for MAO-A than for MAO-B (in accordance with the preponderant MAO-A on MAO-B in human WAT ([24]). Hence, this is the first time, at least to our knowledge, that data evidence the selectivity of harmine towards MAO relative to SSAO inhibition, as this β-carboline was inhibiting the production of neosynthesized radiolabeled benzaldehyde up to 50% only in the millimolar range whereas it deeply inhibited tyramine oxidation at 100 nM. Thus, already described as binding to MAO [44] and to imidazoline I_2_-sites [47], harmine is also able to interact with SSAO to a lesser extent, which is not so astonishing because I_2_-sites have been described on SSAO [48,49].

As noted above, the oxidation of amine substrates generates hydrogen peroxide, irrespective of the amine oxidase catalyzing the oxidative deamination. It was of interest to study the influence of opipramol and of antipsychotics on the fate of H_2_O_2_ in human adipose tissue because the adverse effects of the latter have been reported to be mitigated by antioxidants [50] or AMP-activated protein kinase (AMPK) activators [51].

### 3.5. Opipramol Interacted with Hydrogen Peroxide Generation by Amine Oxidases

**Figure 8:**
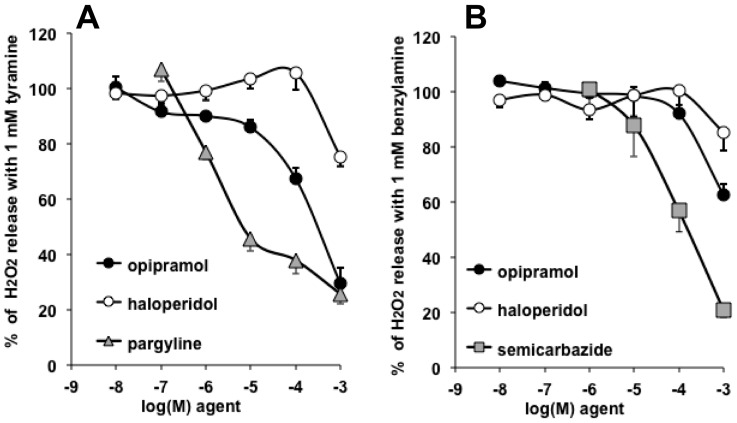
Dose-dependent inhibition by psychoactive agents of the hydrogen peroxide release by human adipose tissue homogenates incubated in the presence of tyramine (**A**) or benzylamine (**B**). The positive controls consisted in measuring the production of hydrogen peroxide by homogenates in the presence of 1 mM of each amine for 30 min, and were set at 100%. Mean ± SEM of seven to eight observations for tyramine and benzylamine, performed without or with increasing doses of haloperidol (open circles), opipramol (closed circles), pargyline as a reference MAO inhibitor (shaded triangles, (A)), and semicarbazide as a reference semicarbazide-sensitive amine oxidase (SSAO) inhibitor (shaded squares, (B)). In several cases, SEM error bars lie within the symbol.

Accordingly with the observations made on [^14^C]-tyramine oxidation, opipramol inhibited MAO activity in hScAT—the fluorometric determination of H_2_O_2_ released during amine oxidation indicated that the anxiolytic agent reached the same level of inhibition as pargyline (Figure 8A). By contrast, only supra-therapeutical, millimolar doses of haloperidol inhibited tyramine oxidation, without exceeding 20% inhibition. Such apparent controversy did not appear when considering their capacities to impair benzylamine-induced H_2_O_2_ production. Yet, the two agents were weak SSAO inhibitors, whereas semicarbazide was as inhibitory as it was with the radiometric method.

Several commentaries are needed to further describe these fluorescence-based experiments.

First, phenelzine was not included in the comparative approach, because at doses higher than 10 µM, it generates an autofluorescent signal that impairs hydrogen peroxide detection. This was not the case for pargyline, haloperidol, opipramol, and semicarbazide—when they were present at 1 mM, the readouts of fluorescence represented 95 ± 2%, 95 ± 2%, 87 ± 3%, and 105 ± 3% of 5 µM hydrogen peroxide signal, respectively (*n* = 6–12).

Second, the H_2_O_2_ spontaneous release by human adipose tissue, in the absence of added amine, averaged 0.21 ± 0.03 nmol/100 mg protein/min and represented 30% of the levels detected in the presence of tyramine, or 20% of those seen with benzylamine, that is, corresponding to remaining portions when maximal inhibitions were reached by 1 mM of each reference inhibitor, and by opipramol in the case of tyramine (Figure 8). Consequently, it did not appear that the tested agents limited spontaneous hydrogen peroxide release.

Third, the combination of pargyline + semicarbazide (P + S) did not further inhibit the tyramine-induced signal any more than did the MAO-inhibitor pargyline (28 ± 3% vs. 27 ± 4%, NS). In addition, P + S inhibited as much as semicarbazide the benzylamine signal (17 ± 2% vs. 20 ± 3%, NS). This indicated that, alongside baseline H_2_O_2_ production, the tyramine-induced signal was mainly MAO-dependent, whereas the benzylamine signal was predominantly due to SSAO activity.

Fourth, the tendency of 1 mM haloperidol to inhibit hydrogen peroxide release when human adipose tissue was incubated with either benzylamine or tyramine was not due to its vehicle, DMSO (1% v/v final) because more than 93% of the fluorescence signal was detected in the presence of 1% DMSO (not shown). Thus, only a weak tendency to inhibit MAO and SSAO activity can be attributed to the supra-therapeutic doses of this typical antipsychotic.

Lastly, once all these considerations were taken into account, these experiments described noticeable MAO inhibitory properties for opipramol, therefore adding more complexity for the classification of this agent used for the treatment of neuropsychiatric disorders, which does not fit typically in only one of the following groups: antidepressants, antipsychotics, or anxiolytics.

### 3.6. Lack of Opipramol Effect on Basal or Insulin-Induced Glucose Uptake in Human Adipocytes

Besides lipid mobilization, the incorporation of glucose into lipids is a pathway by which adipocytes manage their size and energy stores. In a search for other direct actions of opipramol on fat cells, it was tempting to verify whether the pharmaceutical agent could alter glucose transport, especially its component highly regulated by insulin. The human adipocyte preparations used in these experiments were not tested in parallel, and exhibited different insulin responsiveness regarding activation of hexose uptake, as in one set, the basal glucose was hardly doubled compared to baseline, whereas in the other, the maximal response to insulin reached an approximately fourfold increase over basal uptake (Table 1). Nonetheless, neither opipramol nor phenelzine was able to activate uptake or to alter insulin stimulation of glucose transport (Table 1). However, phenelzine, owing to its capacity to abolish SSAO activity, inhibited the glucose transport activation by benzylamine, already documented to mimic insulin effect in an H_2_O_2_-dependent and SSAO-mediated manner [27].

Such observation of a lack of direct interplay between opipramol or phenelzine and glucose transport is in agreement with previous observations [6], and contrasts with the direct impairment of insulin signaling reported for olanzapine in muscle cells [52]. Whether the psychoactive drugs act differently on the glucose transport in adipose depots than in muscles or in other tissues is highly conceivable, at least regarding to the discrepancies observed rats after chronic treatment with olanzapine treatment—induction of insulin resistance without alteration of insulin stimulation of glucose transport in adipocytes [10].

## 4. Conclusions

Olanzapine, which is an antipsychotic well-recognized for its adverse effects on weight gain and glucose homeostasis, was not the worst of the diverse pharmaceutical agents tested on lipolytic activity of adipocytes. In the present study, this molecule did not acutely impair lipolysis regulation in human fat cells, although it has been previously demonstrated to alter lipolytic responses after prolonged treatments in rodents or in cultured preadipocytes [11,12,15]. Besides interspecific differences, it seems that it is the mode of action of olzanzapine that underlies such discrepancies. The drug probably more dramatically alters the gene regulation of key factors involved in the triglyceride metabolism (hormone-sensitive lipase, fatty acid synthase) than the short-term regulation of enzyme activities involved in lipolysis. Facing to this lack of novel insight regarding olanzapine adverse effects, our study evidenced unexpected properties of other antidepressant drugs, as highlighted below.

Phenelzine is an antidepressant drug considered as a MAO inhibitor, which also inhibits SSAO, renamed primary amine oxidase or vascular adhesion protein-1 (SSAO/PrAO/VAP-1) [45]. Although it is associated with a limited body weight gain in treated depressed patients [41], we have recently reported that it can improve metabolic control and limit fat accretion in mouse models [8,9]. Here, we report its impressive capacity to inhibit human SSAO at a submicromolar dose. These findings are therefore totally confirmatory of a previous report accumulating three independent approaches showing that phenelzine strongly inhibits SSAO: pharmacological high-throughput screening, enzyme kinetics, and computational docking [53]. Moreover, the present findings bring evidence that phenelzine direct action on adipocytes affects the glucose transport dependent on biogenic amine degradation, as well as its related hydrogen peroxide handling, rather than the lipolytic response to catecholamines. Contrarily to olanzapine, such a drug is not considered to hamper insulin sensitivity, and this was verified in our limited model, as it did not alter short-term insulin activation of glucose uptake in fat cells. Initially considered as only a “protective” agent allowing a longer half-life for catecholamines and facilitating their postsynaptic actions, this multipurpose molecule still deserves further studies and remains a good candidate for drug repurposing, as is the case for olanzapine, recently proposed for neurodegenerative diseases with body weight loss [54]. In this view, its direct antiadipogenic [7] and antilipogenic [6] actions on adipose cells should be taken into account, as well as its carbonyl scavenger properties and its interactions with the gamma-aminobutyric acid (GABA)-ergic system [22,55].

We serendipitously observed that opipramol inhibits the lipolytic activation by cAMP- or cGMP-dependent pathways. Among the diverse psychoactive drugs tested in this study, only risperidone partially reproduced such an antilipolytic effect. Surprisingly, opipramol treatment is not associated with adverse body weight gain, as is the case for olanzapine. One can assert that the in vitro effects of these drugs are not predictive of their therapeutic actions because they were mainly observed at supra-therapeutic levels. However, opipramol is administered at doses up to 200 mg/day [23,38], making our observations at 1–10 µM relevant. Moreover, risperidone, which induces a modest antilipolytic effect, also modestly increases adiponectin levels in treated patients with schizophrenia, wheraes olanzapine decreases the circulating levels of this insulin-sensitizing adipokine [56] and increases pro-inflammatory cytokines [57]. This allows the supposition that opipramol could also have opposite effects to olanzapine when considering adipocyte functions other than lipolysis and glucose transport. Thus, lipogenic responses and adipokine secretion deserve to be further studied with opipramol. Evidently, other central and peripheral actions of these pharmacological agents may entirely explain their different influence on adipose tissue development. In this view, it can be hypothesized than the antilipolytic properties of opipramol might be accompanied by a protective action towards the onset of insulin resistance. As proposed for other antilipolytic agents [58], such potential benefits may occur by limiting the deleterious consequences of excessive circulating lipids, also known as lipotoxicity, which contributes to the complications of various metabolic diseases.

## Figures and Tables

**Table 1:** Glucose transport in human adipocytes.

	Hexose Uptake into Fat Cells (Fold Increase of Basal 2-DG Uptake)			
	Experiment 1		Experiment 2	
	Control	+Opipramol	Control	+Phenelzine
basal	1.00 ± 0.16	1.12 ± 0.14	1.00 ± 0.15	1.09 ± 0.15
insulin 10 nM	2.05 ± 0.38 *	2.12 ± 0.55 *	3.18 ± 0.42 **	ND
insulin 100 nM	2.66 ± 0.29 **	2.41 ± 0.47 **	3.91 ± 0.55 **	4.09 ± 0.63 **
benzylamine 1 mM	ND	ND	1.60 ± 0.14 *	1.03 ± 0.20 #

2-Deoxyglucose (2-DG) uptake was assayed on 10 min after 45 min incubation with the indicated agents, in the absence (control) or in the presence of 100 µM of opipramol (experiment 1) or phenelzine (experiment 2). Values are means ± SEM of 4 and 10 individuals for experiment 1 and 2, respectively. Different from respective basal at *: *p* < 0.05; **: *p* < 0.01. Different from corresponding control at #: *p* < 0.05. ND: not determined.
